# Identification of Two Eosinophil Subsets in Induced Sputum from Patients with Allergic Asthma According to CD15 and CD66b Expression

**DOI:** 10.3390/ijerph192013400

**Published:** 2022-10-17

**Authors:** Elena Curto, Éder F. Mateus-Medina, Astrid Crespo-Lessmann, Rubén Osuna-Gómez, Cristina Ujaldón-Miró, Alba García-Moral, Paula Galván-Blasco, Lorena Soto-Retes, David Ramos-Barbón, Vicente Plaza

**Affiliations:** 1 Asthma Unit, Respiratory and Allergy Department, Hospital de la Santa Creu i Sant Pau, Biomedical Research Institute Sant Pau (IIB Sant Pau), Department of Medicine, Universitat Autònoma de Barcelona, 08041 Barcelona, Spain; 2 Inflammatory Diseases Unit, Biomedical Research Institute Sant Pau (IIB Sant Pau), 08041 Barcelona, Spain; 3 Cellular Immunotherapy and Gene Therapy Group (GITG), Oncology, Hematology and Transplantation Laboratory, Biomedical Research Institute Sant Pau (IIB Sant Pau), 08041 Barcelona, Spain; 4 Pediatric Allergy Unit, Pediatric Allergy Section, Pediatric Pneumology and Cystic Fibrosis, Pediatrics Service, Hospital Universitari Vall d’Hebron, 08041 Barcelona, Spain; 5 Allergology Section, Department of Internal Medicine, Hospital Universitari Vall d’Hebron, 08041 Barcelona, Spain

**Keywords:** allergic asthma, eosinophil subsets, flow cytometry, induced sputum

## Abstract

Two subsets of eosinophils have been described: resident eosinophils with homeostatic functions (rEOS) in healthy subjects and in patients with nonallergic eosinophilic asthma, and inflammatory eosinophils (iEOS) in blood and lung samples from patients with allergic asthma. We explored if it would be possible to identify different subsets of eosinophils using flow cytometry and the gating strategy applied to induced sputum. We conducted an observational cross-sectional single-center study of 62 patients with persistent allergic asthma. Inflammatory cells from induced sputum samples were counted by light microscopy and flow cytometry, and cytokine levels in the supernatant were determined. Two subsets of eosinophils were defined that we call E1 (CD66b-high and CD15-high) and E2 (CD66b-low and CD15-low). Of the 62 patients, 24 were eosinophilic, 18 mixed, 10 paucigranulocytic, and 10 neutrophilic. E1 predominated over E2 in the eosinophilic and mixed patients (20.86% vs. 6.27% and 14.42% vs. 4.31%, respectively), while E1 and E2 were similar for neutrophilic and paucigranulocytic patients. E1 correlated with IL-5, fractional exhaled nitric oxide, and blood eosinophils. While eosinophil subsets have been identified for asthma in blood, we have shown that they can also be identified in induced sputum.

## 1. Introduction

Asthma clinical practice guidelines [1,2] recommend phenotyping patients with severe asthma to guide decisions on biological treatment. The most widely used method to establish phenotype is blood eosinophil count based on a 300 cells/µL cut-off. In patients with severe asthma and <300 eosinophils/µL in blood (around 30% of cases), induced sputum can be used to diagnose bronchial eosinophilia [3,4], coexistent or isolated neutrophilic inflammation, and pauci-inflammatory profiles. While counting induced sputum cells using light microscopy is an effective means of identifying bronchial leukocytes, this approach is both laborious and requires specially trained professionals.

Flow cytometry, using specific markers for membrane proteins, can also be used to automatically count cells in induced sputum. Although this application has been described [5,6], it has not yet been fully standardized. In blood and other biological fluids, flow cytometry can classify leukocytes and correctly identify eosinophils [7] and can even differentiate between eosinophil subsets with different functions in several organs and systems [8,9,10]. In addition to the eosinophils known to play an inflammatory role in defense against helminths (called inflammatory eosinophils, or iEOSs), there are other eosinophils with homeostatic functions and a long half-life that reside in tissues (called resident eosinophils, or rEOSs) [11,12]. These two eosinophil subsets have been identified in the mouse lung, where they are recruited after exposure to an allergen [13]. Both subsets have also been identified in human samples, with iEOS predominating in nonallergic eosinophilic asthma, and rEOS in allergic asthma and healthy controls. While rEOSs generally have a greater adhesion capacity, both subsets show greater survival in nonallergic eosinophilic patients than in allergic or healthy patients [14]. These two subsets have been also identified in blood and nasal polyps of patients with severe eosinophilic asthma plus concomitant chronic rhinosinusitis with nasal polyps [15]. All those studies used CD62L to differentiate between eosinophil subsets [13,14,15]. CD62L, an adhesion molecule present in multiple blood cells, is shed from the eosinophil membrane after passage through the endothelium. Since CD62 detection in induced sputum is predictably low [16], a different gating strategy is needed.

Evidence on the existence of different eosinophil subsets may have clinical implications for phenotype definition, patient prognosis, and treatment. Identifying eosinophil subsets in patients with severe, uncontrolled asthma is likely to enable judicious selection of biological treatments; it may even help identify targets for the development of new molecules and treatments specifically focused on pathological eosinophils, which would avoid the deleterious effects of concomitantly eliminating eosinophils with homeostatic functions.

The objective of this study was to explore the possibility of distinguishing between eosinophil subsets in the induced sputum of patients with allergic asthma and to determine the distribution of these subsets in different bronchial inflammatory phenotypes.

## 2. Materials and Methods

In this single-center cross-sectional observational study, patients monitored for persistent allergic asthma were recruited from Hospital de la Santa Creu i Sant Pau outpatient clinics between September 2016 and January 2019. All patients met diagnostic criteria for persistent asthma according to the current version of the Global Initiative for Asthma (GINA) [1], were in maintenance treatment with inhaled corticosteroids (ICS), and signed a written informed consent. Excluded were patients with other serious respiratory diseases, on chronic corticosteroid or monoclonal antibody treatment for asthma, or who did not provide informed consent. Allergic asthma was defined as a positive skin prick test for common aeroallergens or a positive allergen-specific immunoglobulin E (IgE) test and respiratory symptoms after exposure to the allergen. Data compiled from the latest available analysis included blood eosinophil count and total IgE, exacerbations in the previous year that required systemic corticosteroids, and any maintenance treatment administered. Spirometry and fractional exhaled nitric oxide (FeNO) using Evernoa BASE (EverSens) were performed. The Spanish-validated version of the Asthma Control Test (ACT) [17] was administered to all patients. Patients’ induced sputum was analyzed by microscopy and flow cytometry, and cytokines present in the supernatant were analyzed.

### 2.1. Sputum Induction and Processing

Sputum induction was performed according to the European Respiratory Society (ERS) standardized methodology [18]. Premedication with 200 mg of inhaled salbutamol was followed by nebulization of a hypertonic solution with an ultrasonic nebulizer (NE-007, Omron, Kyoto, Japan). Nebulizations lasted seven minutes each and consisted of hypertonic saline at increasing concentrations (3%, 4%, and 5%) until an adequate amount of sputum was obtained. The sputum was processed within two hours of collection, and mucus plugs were selected for treatment with dithiothreitol (Sputalysin, Calbiochem Corp., San Diego, CA, USA) and phosphate buffered saline (PBS). The cell suspension was then filtered to remove detritus. Number of cells per gram of sputum, cell viability, and total squamous cells from contaminated upper respiratory tracts were calculated using a hemocytometer and trypan blue staining. Cell preparations were centrifuged to obtain cell pellets and supernatant. The cells’ pellets were used for both differential cell counting (using Wright–Giemsa staining) and population analysis (using flow cytometry), and the supernatant was frozen at −80 °C until further analysis. Following ERS recommendations [18], patients were classified by bronchial inflammatory phenotype as follows: paucigranulocytic (eosinophils < 3%, neutrophils < 65%), neutrophilic (eosinophils < 3%, neutrophils ≥ 65%), eosinophilic (eosinophils ≥ 3%, neutrophils < 65%), and mixed (eosinophils ≥ 3%, neutrophils ≥ 65%) [19].

### 2.2. Flow Cytometry

Leukocytes were identified using autofluorescence (FITC), side scatter (SSC), and CD45 expression [5]. Expression of eosinophil surface markers, activation markers, and characteristic cell function markers was determined (CD66b, CD16, CD15, CD62L, CD63, CCR3, CD123, CD125, and Siglec-8-9). Cells were incubated at 4 °C in darkness with different combinations of antibodies. After 20 min, they were washed with PBS + 0.5% bovine albumin and then resuspended in 200 µL of PBS. Samples in which cell viability was ≥80% and >10,000 cells could be analyzed per determination were analyzed in a MACSQuant 10 cytometer (Miltenyi Biotec, Bergisch Gladbach, Germany). FlowJoX v10.7 software was used to analyze expression levels and the percentage of positive cells for each marker in viable eosinophils, identified based on size and granularity parameters in combination with CD45+, CD66b+, CD16−, and CD15+ expression.

**Figure 1:**
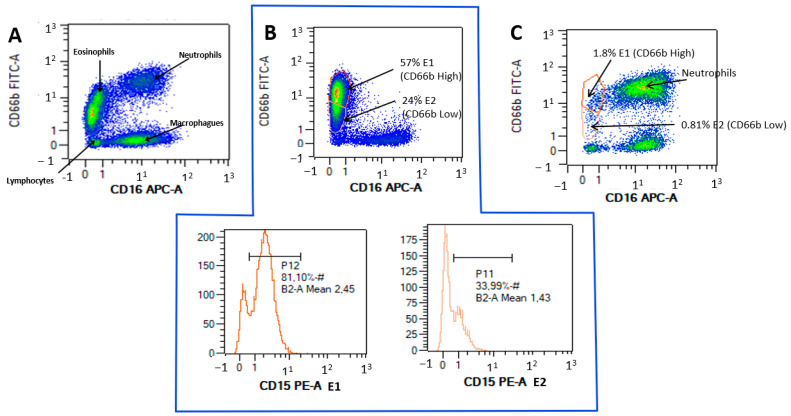
(**A**): Flow cytometry for a patient presenting all cell lines, based on CD16 APC-A and CD66B FITC-A. (**B**): A highly eosinophilic patient with E1 predominance over E2 (57% vs. 24%), with side plots showing CD15 expression of 81% for E1 and 33.99% for E2. (**C**): A neutrophilic patient, with few E1 eosinophils (1.8%) or E2 eosinophils (0.8%).

Flow cytometry gating defined the eosinophil population that coexpressed CD66b+ and CD15+. Two eosinophil subsets, which we call E1 and E2, were characterized by high expression of CD66b and CD15 and low expression of CD66b and CD15, respectively. Figure 1 shows flow cytometry examples of eosinophil populations in the induced sputum of patients with allergic asthma.

### 2.3. Supernatant Cytokine Analysis

Cytokines present in the induced sputum supernatant were analyzed, using LEGENDplex panels (BioLegend, San Diego, CA, USA), to determine levels of the following: eotaxin; immunoglobulins IgA, IgD, IgE, IgG1, IgG2, IgG3, IgG4, and IgM; interferon alpha and gamma (IFNα and IFNɣ); interleukins IL-1Β, IL-4, IL-5, IL-6, IL-8, IL-10, IL-12p70, IL-13, IL-17A, IL-18, IL-23, and IL-33; monocyte chemoattractant protein 1 (MCP-1); matrix metalloproteinase 2 and 9 (MMP2 and MMP9); and tumor necrosis factor alpha (TNFα).

### 2.4. Statistical Analysis

For normally distributed continuous variables, results were expressed as means and standard deviations (SD), and qualitative variables were expressed as frequencies and percentages. Differences between phenotypes were determined using analysis of variance (ANOVA) for the quantitative variables and Pearson’s chi-squared for the qualitative variables. Posthoc analyses were performed using the Sheffe and Games Howell tests for homogeneity and nonhomogeneity of variances, respectively. Pearson’s correlation coefficient was used to determine dependence between quantitative variables. In all cases, the level of statistical significance was set to 5% (α = 0.05). Statistical analysis was performed using SPSS V. 24.0 for Windows.

## 3. Results

Clinical characteristics for the 62 recruited patients with allergic asthma are summarized in Table 1. Mean (SD) values in induced sputum were as follows: by microscopy, eosinophils 12.27% (16.71%), and neutrophils 58.85% (18.32%); and by flow cytometry (Table 2), eosinophils 18.59% (22.56%), and neutrophils 63.05% (25.21%). Flow cytometry measured a mean (SD) of 14.24% (19.79%) for E1 and of 4.71% (6.54%) for E2. E1 correlated with FeNO (r = 0.357; *p* = 0.050) and blood eosinophils (r = 0.382; *p* = 0.003), and E2 with the ACT score (r = 0.388; *p* = 0.021). E1 and E2 correlations with exacerbations did not reach statistical significance.

Bronchial inflammatory phenotype distribution, according to induced sputum cellularity as measured by microscopy, were as follows (Table 1): eosinophilic, 24 patients (37.5%); mixed, 18 patients (28.12%); and neutrophilic or paucigranulocytic, each 10 patients (15.62%). Neutrophilic patients showed a higher incidence of smokers and exsmokers (*p* = 0.040); paucigranulocytic patients, lower ICS requirements (*p* = 0.033); and eosinophilic and mixed patients, a higher incidence of nasal polyposis (*p* = 0.042).

**Figure 2:**
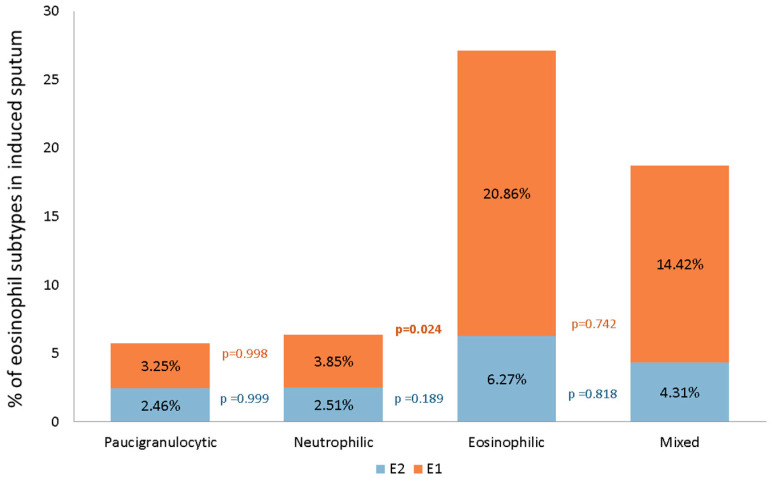
E1 and E2 distributions according to different bronchial inflammatory phenotypes.

All four phenotypes had detectable E1 and E2 subsets. E2 was similar across the different phenotypes (*p* = 0.302), whereas E1 differed (*p* = 0.034). E1 predominated over E2 in eosinophilic (20.86% vs. 6.27%) and mixed (14.42% vs. 4.31%) patients, while E1 and E2 distributions were similar for neutrophilic (2.51% vs. 3.85%) and paucigranulocytic (3.25% vs. 2.46%) patients (Figure 2). Posthoc analysis of E1 confirmed differences between neutrophilic and eosinophilic patients (3.85% vs. 20.86%; *p* = 0.024) and between paucigranulocytic and eosinophilic patients (3.85% vs. 20.86%; *p* = 0.020).

No statistically significant differences between phenotypes were evident from supernatant cytokines (Table 2). There were significant correlations in the overall sample for IL-5 with certain parameters, specifically, eosinophils by microscopy and flow cytometry (r = 0.885; *p* = 0.019 and r = 0.975; *p* = 0.001, respectively), inversely with neutrophils by microscopy (r = −0.858; *p* = 0.029), and with E1 (r = 0.971; *p* = 0.001; see Supplementary Material Figure S1) but not E2 (r = 0.571; *p* = 0.232).

## 4. Discussion

In this exploratory study we demonstrate that, using flow cytometry, it is possible to identify two subsets of eosinophils in induced sputum in patients with persistent allergic asthma. Subset E1, characterized by high expression of CD66b and CD15, predominates over subset E2 in allergic asthma with bronchial eosinophilic inflammation. E1 levels are correlated with FeNO, blood eosinophil count, and IL-5 levels in induced sputum supernatant, which would suggest that they play more of an inflammatory than a homeostatic role.

As this is the first study that defines subsets of eosinophils in induced sputum, there were no precedents available. For this reason, our gating strategy relied on the expression of CD66b and CD15. The role of CD66b, a membrane protein present in granulocytes that indicates cell activation, in eosinophils has only recently been described. Its activation by monoclonal antibodies or its usual ligand, galectin-3, induces cell adhesion, superoxide molecule production, and eosinophil degranulation [20]. CD15 (also called Lewis X antigen or SSEA-1) is a carbohydrate forming part of the adhesion molecules present in cell membranes, mainly neutrophils but also eosinophils [21]. Its role in eosinophils is not, as yet, well-understood, although a study of patients with hypereosinophilic syndrome has shown that CD15 induces eosinophil cationic protein release and therefore plays a role in tissue damage associated with this syndrome [22]. Expression of CD66b and CD15 was elevated in the E1 subset, probably reflecting an activated cellular state. In studies performed in blood [13,14], iEOS was shown to decrease rapidly after specific bronchoprovocation14, theoretically due to recruitment to the airways. E1 predominance in asthma with eosinophilic inflammation, molecule expression in its membrane, and the link with FeNO and IL-5 may lead to the conclusion that E1 are the iEOS referred to in other studies, even if the latter are defined by other flow cytometry cell markers; however, such a conclusion requires further investigation.

Studies comparing techniques such as induced sputum and bronchoalveolar lavage for evaluation of airway eosinophils have reported weak correlations [23,24], probably because the techniques evaluate different airway zones (bronchial lumen, subepithelial compartment, and intraepithelial compartment). Since a recent study shows that induced sputum eosinophils correlate with subepithelial eosinophils concentrations [25], it would be interesting to complement that research with bronchial biopsies to assess whether the two subsets are also present and correlated in the subepithelium and the bronchi. Although it may be thought that the two subsets correspond to different maturation stages, irrespective of location, bronchial eosinophils show a certain degree of activation as a consequence of exposure to inflammatory mediators and expression of membrane proteins as required to migrate [26].

Except for a recently published study in mice [27], the available evidence points to iEOS being the only subset dependent on IL-5 [13]. This is important, as treatment with anti-IL-5 or anti-IL-5Ra can influence the two subsets in a different way, probably eliminating iEOS without affecting rEOS. Understanding the functions of these eosinophil subsets and identifying them in patients before starting biological therapies may be decisive in the choice of monoclonal antibody [28] or the evaluation of lack of therapeutic response.

For a large series of patients with differing asthma severity levels, the distribution of inflammatory phenotypes in induced sputum was reported as eosinophilic 37–43%, paucigranulocytic 37–45%, neutrophilic 15–16%, and mixed 3–4% [29]. However, it is not known whether those percentages are reflected in allergic asthma populations such as ours. Allergic asthma is usually accompanied by elevated FeNO and blood eosinophil levels and is generally considered to be eosinophilic [30], yet classifications according to blood eosinophils show that allergic asthma may be noneosinophilic in up to 50% of patients with mild–moderate asthma [31], and also in patients with severe asthma who are candidates for omalizumab [32]. Furthermore, some studies report that induced sputum lymphocyte and neutrophil values for allergic asthma are higher than for nonallergic asthma [33] and that, after specific bronchoprovocation, both neutrophils and eosinophils increase significantly in the airways [34]. In our series, a third of allergic patients were noneosinophilic and 45% had raised neutrophil levels. We found this finding novel and interesting, as it may have therapeutic and prognostic implications for patients with allergic asthma.

The absence in our patients of significant differences in the level of cytokines present in induced sputum is probably explained by the small number who presented detectable levels and by the effect of dithiothreitol [35,36].

Some limitations of this study are its single-center nature and limited number of patients, indicating the need for external validation to confirm the results. As investigations for the future, it would be useful to conduct exploratory studies to correlate our findings with data from blood or bronchial samples, and even to perform the analyses before and after bronchoprovocation with allergens. The relationship between chronic ICS treatment and eosinophil populations was not analyzed since we had no data on asthma treatment adherence, not to mention the fact the acknowledged high level of nonadherence means that such data could introduce bias. It would be interesting to compare how E1 and E2 are distributed in the induced sputum of healthy patients and in patients with nonallergic asthma.

Note that the aim of this study was not to relate eosinophil subsets to clinical variables, but to describe the gating strategy applied to induced sputum and the distribution of eosinophil subsets in different types of allergic patients. The population on which this study was based is a pool of patients with persistent asthma of different degrees of severity, and although mainly moderate, we consider this subpopulation capable of reflecting the same local inflammatory processes as severe asthma.

Although different populations of eosinophils have already been defined for many organs and systems, lung and airway studies are scarce, despite both the important role played by these organs in asthma and the availability of targeted treatments. Our study, by providing new evidence on the possibility of identifying eosinophil subsets in induced sputum using a noninvasive technique, opens doors to new lines of research in asthma and other respiratory diseases.

## 5. Conclusions

Different eosinophil subsets can be identified in various organs and systems. For asthma, these subsets have already been identified in blood, but we have shown that they can also be identified in induced sputum. The E1 subset (CD66b-high and CD15-high) predominates in patients with allergic asthma and eosinophilic inflammation and is correlated with blood eosinophils, FeNO, and IL-5 in sputum supernatant.

## Figures and Tables

**Table 1:** Clinical, functional, and light microscopy characteristics based on the induced sputum phenotype.

Variables Mean (SD)/*n* (%)		All (*n* = 62)	Paucigranulocytic (*n* = 10)	Neutrophilic (*n* = 10)	Eosinophilic (*n* = 24)	Mixed (*n* = 18)	*p*
General characteristics	Age, years	51.40 (10.79)	41.80 (12.06)	53.30 (11.02)	52.38 (9.94)	54.39 (8.69)	**0.017**
	Sex, female	29 (48.3%)	7 (70%)	6 (60%)	9 (37.5%)	8 (44.4%)	0.300
	BMI, kg/m^2^	28.60 (5.52)	25.57 (5.62)	27.76 (5.44)	30.05 (6.19)	28.82 (4.06)	0.177
	Never smoked	42 (70%)	7 (70%)	4 (40%)	19 (79.2%)	14 (77.8%)	**0.044**
	Active smoker	4 (6%)	2 (20%)	0	1 (4.2%)	1 (5.6%)	
	ICS dose, low	18 (30)	6 (60%)	0 (0%)	6 (25%)	6 (33.3%)	**0.033**
	ICS dose, medium	27 (45%)	3 (30%)	5 (50%)	12 (50%)	8 (44.4%)	
	ICS dose, high	15 (25%)	1 (10%)	5 (50%)	6 (25%)	4 (22.2%)	
	Exacerbations in the previous year	1.15 (1.55)	0.90 (0.99)	1.00 (1.05)	0.83 (0.91)	1.78 (2.41)	0.236
	ACT score	18.82 (7.52)	17.67 (6.68)	14.60 (5.63)	20.50 (8.47)	19.00 (7.04)	0.490
Comorbidities	Rhinitis	44 (73.3%)	9 (90%)	5 (50%)	19 (79.2%)	14 (75.8%)	0.178
	Nasal polyps	16 (26.7%)	1(10%)	0 (0%)	7 (29.2%)	8 (44.4%)	**0.042**
Blood test	Eosinophils (cells/mm^3^)	352.5 (328.8)	142 (137.5)	255 (228.78)	430.4 (300.37)	428.1 (438.77)	0.062
	Total IgE (U/L)	269.56 (415.38)	141.43 (138.18)	169.04 (138.10)	250.90 (306.41)	436.61 (683.06)	0.282
Lung function	FEV_1_/FVC	66.50 (13.78)	74.80 (8.70)	62.54 (14.11)	69.41 (15.00)	64.05 (12.81)	0.143
	FEV_1_ (% ref)	80.36 (22.73)	93.70 (13.31)	80.83 (22.55)	78.97 (21.88)	75.44 (25.44)	0.212
	FEV_1_ (L)	2.49 (0.88)	2.99 (0.75)	2.30 (0.75)	2.50 (0.94)	2.36 (0.87)	0.252
	FeNO (ppb)	37.58 (33.03)	37.21 (30.64)	26.22 (11.82)	42.14 (35.08)	38.04 (39.92)	0.479
Microscopy induced sputum	Eosinophils, %	11.71 (16.48)	0.80 (0.63)	1.15 (0.66)	22.70 (21.66)	9.00 (5.07)	**0.000**
	Neutrophils, %	58.47 (18.60)	42.00 (15.92)	76.65 (10.19)	48.45 (16.68)	70.88 (4.17)	**0.000**
	Macrophages, %	27.44 (17.33)	54.80 (15.58)	20.00 (9.95)	26.04 (15.14)	18.22 (4.64)	**0.000**
	Bronchial cells, %	2.06 (0.62)	2.30 (0.48)	1.60 (0.69)	2.25 (0.44)	1.94 (0.72)	**0.017**
	Lymphocytes, %	1.79 (0.72)	1.90 (0.56)	1.65 (0.66)	1.87 (0.74)	1.72 (0.82)	0.787

Abbreviations: ACT, asthma control test; BMI, body mass index; FeNO, exhaled fraction of nitric oxide; FEV_1_, forced expiratory volume in the first second; FVC, forced vital capacity; ICS, inhaled corticosteroids; IgE, immunoglobulin E; SD, standard deviation. The bold numbers are the ones that reached statistical significance.

**Table 2:** Flow cytometry results: eosinophil populations, phenotypes, and cytokines in induced sputum supernatant.

Variables Mean (SD)/*n* (%)		All (*n* = 62)	Paucigranulocytic (*n* = 10)	Neutrophilic (*n* = 10)	Eosinophilic (*n* = 24)	Mixed (*n* = 18)	*p*
Flow cytometry	Eosinophils, %	17.75 (22.30)	5.73 (10.21)	6.78 (9.08)	25.93 (27.16)	18.94 (20.53)	**0.036**
	Neutrophils, %	64.36 (25.26)	63.05 (27.49)	78.82 (13.66)	56.52 (27.99)	67.45 (22.72)	0.115
	Macrophages, %	0.42 (0.48)	0.51 (0.44)	0.40 (0.30)	0.46 (0.64)	0.34 (0.34)	0.812
	Lymphocytes, %	6.46 (6.94)	12.49 (7.91)	3.17 (1.53)	6.65 (8.19)	5.01 (4.56)	**0.016**
Phenotypes	E1, %	13.57 (19.51)	3.25 (7.58)	3.85 (7.59)	20.86 (24.91)	14.42 (16.11)	0.034 *
	E2, %	4.52 (6.44)	2.46 (3.13)	2.51 (2.58)	6.27 (8.00)	4.31 (6.52)	0.302
	E1 CD15	55.13 (39.92)	66.61 (46.05)	57.70 (35.98)	50.94 (39.29)	53.54 (41.85)	0.791
	E2 CD15	9.69 (17.63)	25.48 (31.61)	6.22 (16.01)	6.65 (11.43)	7.76 (12.60)	0.032 **
Supernatant	IL-5 (pg/mL)	6.83 (4.80)	***	7.44 ****	3.59 (1.86)	8.80 (6.28)	0.604
	IL-4 (pg/mL)	15.50 (17.40)	6.27 (4.92)	9.61 (10.09)	21.90 (23.53)	15.97 (14.15)	0.447
	IL-13 (pg/mL)	8.77 (6.79)	6.13 (3.77)	7.38 (5.12)	11.54 (8.99)	7.21 (4.53)	0.320
	Eotaxin(pg/mL)	15.33 (20.52)	13.80 (13.29)	28.66 (47.42)	12.31 (9.92)	12.67 (7.90)	0.487

Abbreviations: IgE, immunoglobulin E; IL, interleukin; SD, standard deviation. * Differences found between paucigranulocytic and eosinophilic patients (*p* = 0.020) and between neutrophilic and eosinophilic patients (*p* = 0.024). ** No differences found in the posthoc analysis. *** Undetectable. **** Detectable only in one patient. The bold numbers are the ones that reached statistical significance.

## Data Availability

The data that support the findings of this study are available from the corresponding author upon reasonable request.

## Supplementary Materials

The following supporting information can be downloaded at: https://www.mdpi.com/article/10.3390/ijerph192013400/s1, Figure S1: Correlation between IL-5 (pg/mL) and E1 levels (% on total induced sputum eosinophil levels measured by flow cytometry).
