# Supplementary material for: Identification of Two Eosinophil Subsets in Induced Sputum from Patients with Allergic Asthma According to CD15 and CD66b Expression
Source: Int J Environ Res Public Health. 2022 Oct 17;19(20):13400. doi: 10.3390/ijerph192013400 (PMC9602830; doi:10.3390/ijerph192013400)

Supplementary material

Figure S1. Correlation between IL-5 (pg/mL) and E1 levels (% on total induced sputum eosinophil levels measured by flow cytometry).

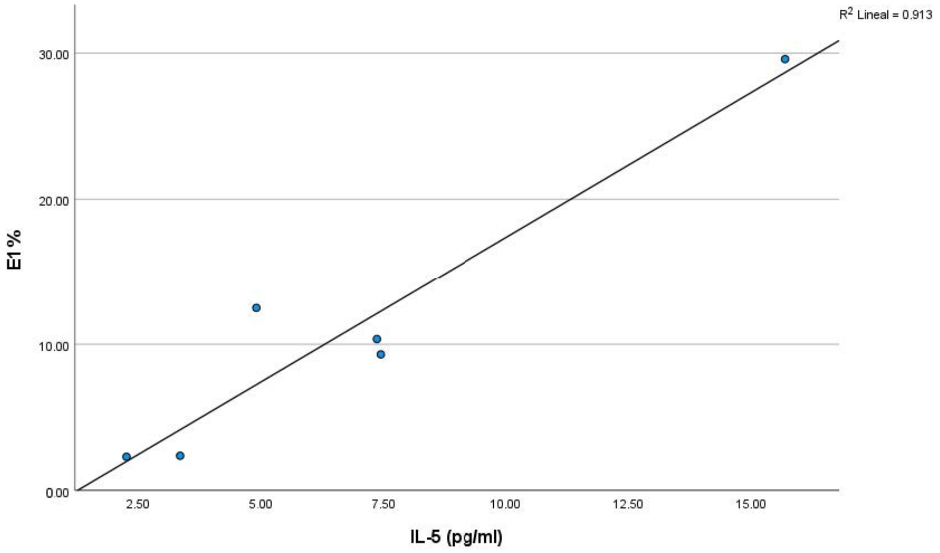

Supplement: Supplementary file 1 [file ijerph-19-13400-s001.zip › ijerph-1905789-supplementary.pdf]
